# Assessing the Adequacy of Orbital Reconstruction With Titanium Mesh Using Clinical and Radiological Measures

**DOI:** 10.7759/cureus.70324

**Published:** 2024-09-27

**Authors:** Kesha Shah, Sangeeta Thakurani

**Affiliations:** 1 Plastic and Reconstructive Surgery, Sawai Man Singh Medical College, Jaipur, IND

**Keywords:** computed tomography, enophthalmos, orbit floor fracture, orbit reconstruction, volumetric analysis

## Abstract

Background

Orbital reconstruction aims to restore the original orbit volume and correct diplopia, enophthalmos, and ocular motility of the fractured orbit. This study aimed to assess the adequacy of orbital reconstruction using radiological and clinical factors.

Methods

In this retrospective study, patients with orbital blowout fractures meeting clinical or radiographic criteria underwent orbital reconstruction with titanium mesh. The orbital volume and anteroposterior displacement were calculated pre- and post-operatively using computed tomography. Diplopia, inferior orbital nerve examination, and ocular movement were also evaluated. Pre- and post-operative orbital volumes of the fractured and contralateral unfractured orbits were compared. Statistical analysis was performed using MS Excel (Redmond, USA) and STATA BE (Texas, US).

Results

There was a significant reduction in the difference in volumes between fractured and normal orbits postoperatively (p-value <0.001). The mean difference between the reconstructed orbital floor fracture and the contralateral normal orbit was 0.55 cm^3^, which is within the normal anatomic variation. Enophthalmos was corrected postoperatively in our patients per radiological parameters as a result of a reduction in the mean posterior displacement. Infraorbital nerve hypoesthesia was not resolved postoperatively.

Conclusion

Our study highlights the restoration of the normal anatomical variation in volume differences between the fractured and contralateral orbits post-surgery using CT-guided analysis, thereby improving clinical outcomes.

## Introduction

The orbital floor and medial wall are frequently involved in trauma and are common fracture sites [1]. The infraorbital nerve traversing within the orbital floor is often involved in orbital floor fracture (OFF). Floor fractures tend to involve the inferior and medial walls, as the lateral part is more resilient to damage [2]. According to most research, the most common mechanism of trauma of OFF is assault among males aged 20 to 30 years, followed by falls and road traffic accidents [3,4].

The hallmark signs of an OFF are diplopia and enophthalmos [5]. Diplopia, or double vision, is a common symptom associated with OFF due to the entrapment of ocular muscles or nerve damage within the fracture site. The disruption of normal eye muscle function or nerve transmission results in misalignment of the eyes, leading to double vision. Enophthalmos can be caused due to various reasons. Early and immediate development of enophthalmos is usually due to an increase in orbital volume due to displacement of the bony framework and herniation of orbital tissue into the maxillary sinus. However, it often develops as a late functional impairment due to scar formation or asymmetric shrinkage of the intraorbital tissues, which becomes difficult to correct and often results in a dissatisfactory final appearance.

When an orbital fracture causes such complications, orbital floor reconstruction is needed [6]. Patients with fractures involving greater than 50% of the orbital floor, an area greater than 1-2 cm^2^, or a herniated mass greater than 1.5 ml have been suggested as surgical candidates [7-9]. The effect of orbital reconstruction and consequent enophthalmos correction can easily be assessed radiologically by calculating the volume and anteroposterior displacement of the reconstructed orbit and clinically by Hertel's exophthalmometer.

This study aims to highlight the importance of integrating radiological and clinical factors in assessing the adequacy of orbital floor reconstruction with titanium mesh.

## Materials and methods

This retrospective study was conducted in the Department of Plastic and Reconstructive Surgery at SMS Hospital, Jaipur, from October 2023 to April 2024. Adult patients admitted with unilateral OFF formed the study population. Patients meeting radiological and clinical criteria for orbital reconstruction, as mentioned below, were included in the study. Patients with bilateral OFF, OFF combined with another facial bone fracture, thyroid-associated ophthalmopathy, direct globe injury, cranial nerve injury, and orbital tumor were excluded from this study.

Radiological and clinical assessment

Measurement of Volume of Bony Orbital Cavity

CT images were taken in the coronal plane with a thickness of 1 mm and in the axial plane with a thickness of 2 mm. The anterior boundary of the orbit was defined as a straight line connecting the anterior lacrimal crest and lateral orbital rim on each axial plane, and the posterior margin was defined as the orbital apex. The volume of the orbit was then calculated by the radiologist. Orbital volumes were measured both pre- and post-operatively and recorded.

Measurement of Enophthalmos

**Figure 1:**
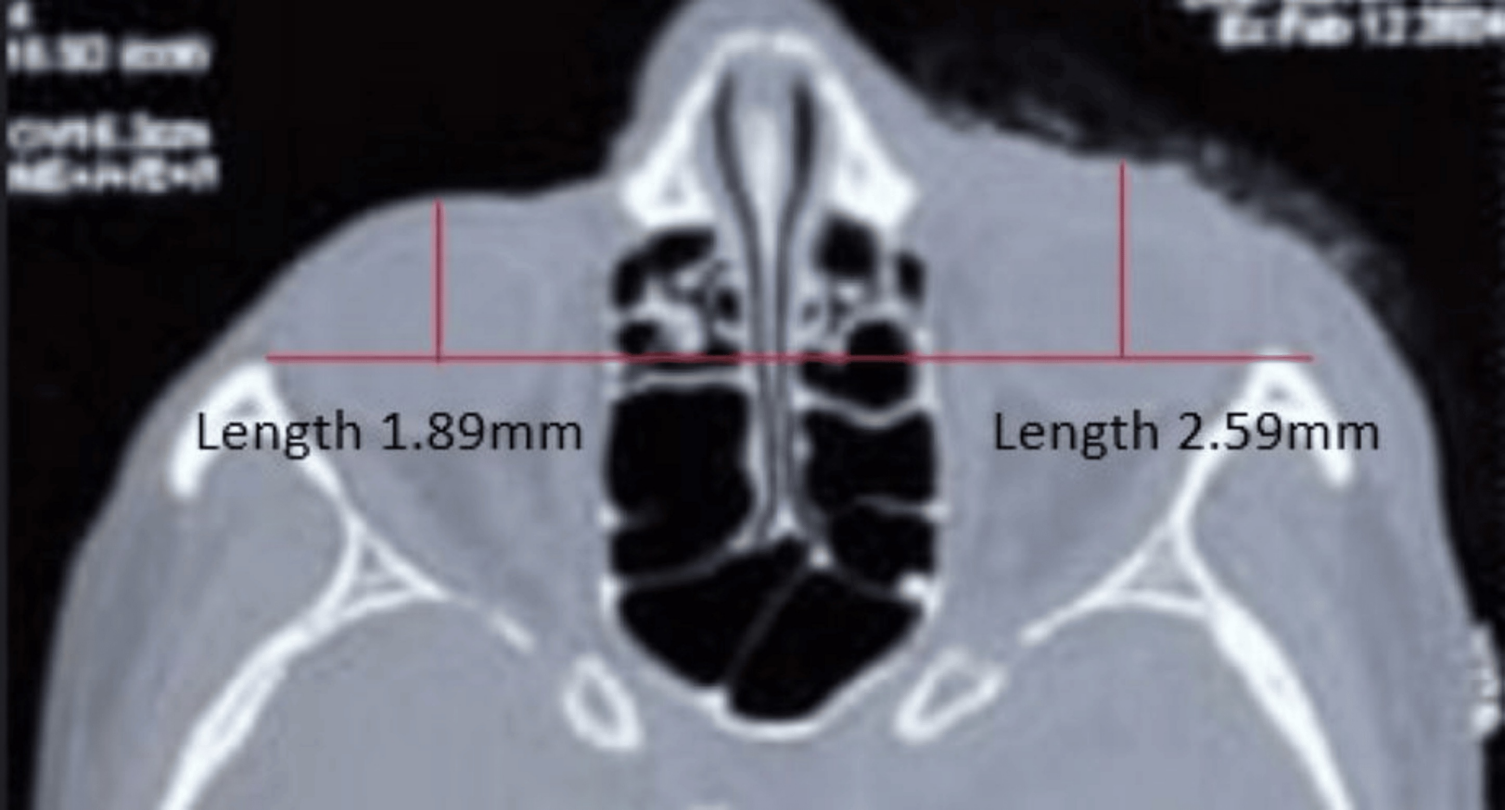
Posterior displacement of the fractured orbit

A horizontal line was drawn to connect the outermost of the bilateral anterior tips of the lateral walls. A vertical line was then drawn from the most prominent corneal part to the baseline. The length of this line was measured as shown in Figure 1. The difference in the measurement value between the normal and the affected orbit was used to conclude the presence or absence of enophthalmos [10,11]. According to Whitehouse et al. (1994), every cm^3^ increase in volume represented approximately 0.77 mm post-traumatic enophthalmos [12]. Thus, radiographic post-traumatic enophthalmos was considered to be present if the measurement was above 0.77 mm. Posterior displacement of the fractured orbit was calculated both pre- and postoperatively and recorded. 

Clinical Criteria

Diplopia was assessed for its persistence or absence. Extraocular movement limitation was assessed using penlight illumination of the cornea with the eye looking in all quadrants. The inferior orbital nerve was also assessed by sensory examination of the lower eyelid, lateral part of the nose, zygoma, and middle part of the upper lip. Enophthalmos was measured clinically, and these measurements were compared with radiological values for posterior displacement of the fractured orbit.

Surgery and postoperative evaluation

Patients who had positive clinical findings (diplopia, restricted extraocular movements, or enophthalmos) or radiologically had an area of defect of orbital floor of more than 2 cm^2^ or an increase in orbital volume of more than 1.3 cm^3^ were included in the study. Surgery was performed by the same surgeon under general anesthesia for all cases. The entire orbital floor defect was exposed through an inferior orbital rim. Herniated orbital contents were reduced and repositioned by a periosteal elevator, a suction tip, and a malleable retractor. OFF was reconstructed using titanium orbital implants (cut to fit the anatomies of fracture sites and soaked in saline mixed with antibiotics) by insertion under the periosteum at fracture sites. We then ensured the absence of restriction using the forced duction test intraoperatively. To reduce orbital tissue swelling, methylprednisolone 250 mg was administered intravenously during surgery and on postoperative days one and two. All patients were discharged on the fourth postoperative day and were followed up till the 14^th^ postoperative day. CT-guided volumetric analysis of the fractured orbit was performed preoperatively and on postoperative day one. These values were then compared with the contralateral normal volume of the orbit.

Since this was a time-based study conducted over a period of six months, the sample size was taken as the number of patients admitted during this time. Statistical analysis was performed using MS Excel and STATA BE software. Continuous data was presented as mean and standard deviation. Categorical data was presented as percentages. Volumes of the fractured and contralateral normal orbits were calculated before and after reconstruction and were compared using the two-tailed Student's t-test.

## Results

Out of the 35 patients diagnosed with OFF, 20 patients had clinical or radiological indications for orbital floor reconstruction. Sixteen out of 20 patients fulfilled the criteria for radiological surgery, as they had a greater than 2 cm^2^ area of defect. The remaining four patients had an area of defect less than 2 cm^2^; however, they met the clinical criteria for surgery. All patients had an increase in orbital volume of more than 1.3 cm^3^. Out of the 20 patients, 10 patients had enophthalmos, two had infraorbital nerve hypoaesthesia and six had restricted ocular movement out of which four had diplopia. The majority of the patients belonged to the age group 41 to 50 years, and 18 (90%) were males. Seventeen (85%) patients were cases of road traffic accidents, two were assault cases, and one patient was a case of falling from height (Table 1). 

**Table 1:** Demographic and clinical information about patients with orbital floor fracture who underwent Titanium mesh reconstruction procedure

Variables	N (%)
Age	44.5 土 9.04 years
Male gender	18 (90%)
Mechanism of injury	
Road traffic accident	17 (85%)
Assault	2 (10%)
Fall from height	1 (5%)
Radiological criteria	
Area of defect >2 cm²	16 (80%)
Increase in volume >1.3 cm³	20 (100%)
Clinical criteria	
Enophthalmos	10 (50%)
Infraorbital nerve hypoesthesia	2 (10%)
Restricted ocular movement with diplopia	4 (20%)
Restricted ocular movement without diplopia	2 (10%)

**Figure 2:**
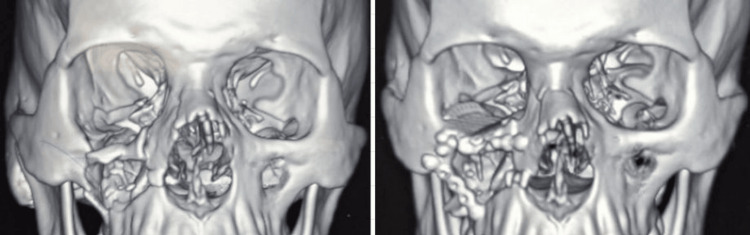
Pre- and post-operative CT image of reconstructed orbit fracture

The difference in volume between the fractured and the unfractured contralateral orbits reduced significantly after reconstruction surgery. The mean volumes of fractured and contralateral normal orbits were measured using CT scans and tabulated (Table 2). According to Tandon et al. (2020), the average anatomical variation between the right and left orbital volumes is 0.8 cm^3^ [13]. Hence, the orbital volume of the fractured orbit was successfully restored after reconstruction, with the asymmetry with the contralateral normal orbit falling within normal anatomical variation (0.55 士 0.42 cm^3^), as shown in Figure 2.

**Table 2:** Volumetric analysis of the fractured and unfractured orbits pre- and post-operatively p-value <0.001 significant for confidence level 99% p-value <0.05 significant for confidence level 95%

	Normal orbit (mean volume, cm3)	Fractured orbit (mean volume, cm3)	Difference of Means (cm3)	p-value
Preoperative	21.41 士 0.54	23.77 士 0.66	2.35 士 0.43	<0.001
Postoperative	21.41 士 0.54	21.88 士 0.71	0.55 士 0.42	0.024
Change in the difference in volumes between fractured and normal orbit postoperatively: 1.80 士 0.13 cm3 (p-value <0.001)				

**Figure 3:**
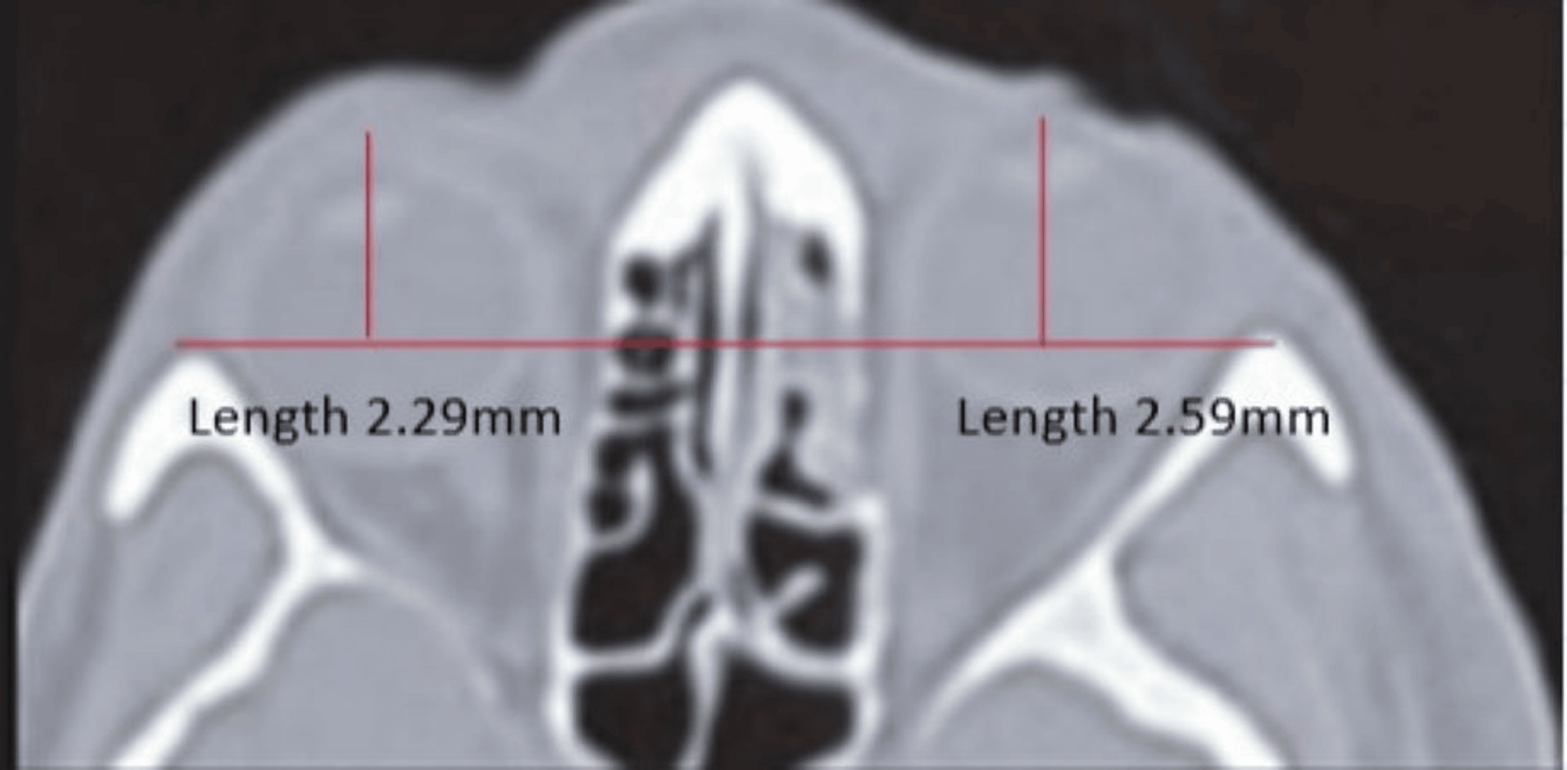
Postoperative posterior displacement of the fractured orbit The difference between the anteroposterior length of the two orbits was reduced to 0.3 mm as compared to 0.7 mm as shown in Figure 1.

In our patients, the mean posterior displacement before reconstruction was 0.93 mm, which was reduced to 0.32 mm postoperatively (Table 3). Whitehouse et al. (1994) radiologically defined enophthalmos as posterior displacements greater than 0.77 mm [12]. Hence, enophthalmos was reversed postoperatively in our patients per radiological parameters, as shown in Figure 3.

**Table 3:** Posterior displacement of fractured orbit ^1^ Enophthalmos present in fractured orbit with posterior displacement of greater than 0.7 mm radiologically

	Posterior Displacement (mm)	Enophthalmos^1^
Pre-operative	0.93	present
Post-operative	0.32	absent

Diplopia and restricted ocular motility were resolved in all of the patients who had these symptoms preoperatively. Enophthalmos, as measured by Hertel's exophthalmometer, was also resolved in all ten patients with preoperative enophthalmos. Loss of sensation in the distribution of the inferior orbital nerve was unfortunately not resolved till the 14^th^ postoperative day.

## Discussion

The aim of orbital reconstruction in orbital fracture is to restore the original volume of the orbit by reducing the herniated contents while also correcting diplopia, enophthalmos, and ocular motility of the fractured orbit. For OFF, many conventional reconstructive options have been employed, such as iliac crest bone and conchal cartilage grafting [14]. In our patients, we used titanium orbital mesh for reconstruction. The gold standard materials for orbital floor reconstruction include titanium implants, which are known for their biocompatibility and durability, and autologous bone grafts, valued for their excellent integration and minimal risk of rejection [15,16]. In this study, we have shown that orbital reconstruction with titanium mesh gives satisfactory results in terms of clinical and radiological factors.

The presence of clinical factors such as diplopia, enophthalmos, or restricted ocular movement in an orbital fracture is an indication of orbital reconstruction. Clinical criteria are often unclear or insignificant. Hence, radiological factors like an increase in the volume of the orbit and posterior displacement of the fractured orbit are used in determining the need for orbital reconstruction. Doing early reconstruction of an orbital fracture prior to the development of clinical symptoms prevents the development of complications such as post-traumatic enophthalmos and fracture malunion [17]. Two out of the 20 patients in this study underwent surgery based on radiological factors in the absence of any clinical indications. Basta et al. (2021) reported that the increase in volume of a fractured orbit is a more reliable indication for surgery as compared to the area of the defect [18]. This contradicted Jin et al. (2000), where the area of defect was used [8]. In our study, all patients had an increase in the volume of more than 1.3 cm^3^, and 16 (80%) patients had an area of defect greater than 2 cm^2^.

Several studies have evaluated the effect of orbital wall reconstruction by calculating volume differences or volume ratios between fractured and contralateral orbits before and after surgery. However, normal anatomic variation between the two orbits was often not taken into consideration [1,19-23]. In our study, we found that the difference in the volume between the fractured and the unfractured contralateral orbit reduced significantly after reconstruction surgery. The mean difference between the reconstructed OFF and the contralateral normal orbit was 0.55 cm^3^, which is within the normal anatomic variation. Enophthalmos was reversed postoperatively in our patients as per radiological parameters as a result of a reduction in the mean posterior displacement.

The retrospective nature of this study and its small sample size are the major limitations of this study. However, the adequacy of titanium mesh reconstruction using clinical and radiological factors has not been studied in this geographic location and population before. The next step would be conducting prospective studies with a longer follow-up time and a larger population cohort.

## Conclusions

This study successfully highlights the importance of integrating radiological and clinical factors in assessing the adequacy of orbital floor reconstruction with titanium mesh. Radiological evidence of increased orbital volume, along with clinical indications of diplopia, enophthalmos, and restricted ocular motility, are important in determining the need for reconstructive surgery. Our study highlights the restoration of the normal anatomical variation in volume differences between the fractured and contralateral orbits postsurgery using CT-guided analysis, thereby reversing enophthalmos and improving clinical outcomes. Prospective studies are required to establish the efficacy of CT-guided radiological analysis in addition to clinical assessment to ensure optimal reconstruction outcomes and to address any residual abnormality.
